# Cholecystomucoclasis: revaluation of safety and validity in aged populations

**DOI:** 10.1186/1471-230X-12-113

**Published:** 2012-08-21

**Authors:** Tomoya Tsukada, Tatsuo Nakano, Takashi Miyata, Shozo Sasaki, Tetsuo Ohta

**Affiliations:** 1 Department of Gastroenterological Surgery, Division of Cancer Medicine, Graduate School of Medical Science, Kanazawa University, 3-1 Takara-machi, Kanazawa, Ishikawa, 920-8641, Japan; 2 Department of Surgery, Asanogawa General Hospital, Kanazawa, Ishikawa, 920-8621, Japan

**Keywords:** Cholecystitis, Cholecystomucoclasis, Deroofing, Subtotal cholecystectomy

## Abstract

**Background:**

We evaluated the safety and validity of cholecystomucoclasis (CM) and compared its intraoperative characteristics with those of standard cholecystectomy (SC).

**Methods:**

We enrolled 174 patients who underwent cholecystectomy and retrospectively evaluated the outcomes of patients in the SC and CM groups.

**Results:**

Significant differences in age (71.1 vs. 61.9 years), American Society of Anesthesiologists physical status (ASA-PS), and serum C-reactive protein levels (CRP) (18.1 vs. 4.7 mg/dL) were observed between the CM and SC groups. Conversely, no significant differences were observed in the operation time (129 vs. 108 min), amount of blood loss (147 vs. 80 mL), intraoperative complications (0% vs. 5.7%), or duration of hospital stay (13.2 vs. 8.9 days) between the 2 groups. A high conversion rate (35.3%), postoperative complications (33%), and frequent drain insertions (94%) were observed in the CM group.

**Conclusions:**

CM is a safe and valid surgical procedure and surgeons should not hesitate to transition to CM for patients who are of advanced age, in poor general condition (high ASA classification), or have high levels of serum CRP.

## Background

Cholecystectomy, particularly laparoscopic cholecystectomy (LC), has become the standard treatment for patients with benign gallbladder disease [1-3]. Although the indications for surgery in patients with acute cholecystitis have been expanding [4-6], there are cases where standard cholecystectomy (SC) is difficult owing to the presence of acute or chronic inflammation, strong omental adhesion, or gangrenous cholecystitis. As patients with benign gallbladder disease tend to be of advanced age, safer and more feasible surgical techniques are required for difficult cases [7-9].

Cholecystomucoclasis (CM) is a traditional method that is combined with subtotal cholecystectomy and involves the cauterization of the posterior gallbladder wall preserved in the liver bed that remains after anterior wall resection, in patients with advanced inflammatory cholecystitis [10]. CM, also called deroofing of the gallbladder [11,12], is a useful procedure that includes partial [13] or subtotal cholecystectomy [14,15]. Recently, increased experience in laparoscopic surgery and other advanced techniques have shown that CM is a safe and feasible option [16-21]. However, preoperative conditions and intraoperative technical characteristics have been less well described. Hence, we revaluated the safety and validity of CM and compared its intraoperative characteristics with those of SC.

## Methods

### Patients

As shown in Table 1, 174 patients (93 men and 81 women) underwent cholecystectomy at the Department of Surgery, Asanogawa General Hospital, Kanazawa, Japan, between January 2007 and December 2011, excluding those who were diagnosed with malignancy and underwent choledocholithotomy or simultaneous resection of other organs.

**Table 1:** Patient characteristics and surgical procedures

	**Number (%)**
Patients	174
Male	93 (53.4)
Female	81 (46.6)
Age (years)	
Mean	62.9±14.3
Range	23–95
ASA-PS classification	
1	39 (22.4)
2	97 (55.7)
3	34 (19.5)
4	4 (2.3)
Surgical procedures	
Cholecystomucoclasis (deroofing)	18 (10.3)
Laparoscopic cholecystectomy (conventional)	163 (93.7)
SILC	18 (10.3)
Open cholecystectomy	11 (6.3)
Conversion (laparoscopic to open)	13 (7.5)

ASA-PS: American Society of Anesthesiologists physical status.

SILC: Single-incision laparoscopic cholecystectomy.

The average age of the patients was 62.9 ± 14.3 years (range, 23–95 years). Preoperative clinical diagnoses of cholecystitis were made on the basis of each patient’s history of right upper abdominal pain and tenderness, fever, leukocytosis, increased C-reactive protein (CRP) levels, and positive signs on computed tomography or ultrasonography (thickened gallbladder wall and pericholecystic fluid collection). All resected specimens were also evaluated histopathologically. This study was approved by the Institutional Review Board of the Asanogawa General Hospital (AGH-IRB No. 22).

### Operative technique

LC was performed by the standard 3 or 4-trocar technique. Briefly, the anesthetized patient was placed in the standard supine, crucifix, reverse-Trendelenburg position, with the surgeon on the patient’s left side. Pneumoperitoneum was achieved by visually guided, cannular CO_2_ insufflation. The dissection was started at the Calot’s triangle, and the cystic duct, the common bile duct, and the cystic artery were exposed and divided between clips. Intraoperative cholangiography was routinely performed to detect residual calculus and bile duct injury. The gallbladder itself was carefully mobilized from the liver bed using electrocautery. An endobag was always used to remove the gallbladder, thus preventing wound infection. The abdominal cavity was irrigated before the trocars were removed, and the fascial defects were closed.

In April 2010, single-incision laparoscopic cholecystectomy (SILC) was instituted at our institution and subsequent LCs were performed using this technique. For the SILC technique, the patient was positioned supine on the operating table with the legs widely separated. The surgeon stood between the patient’s legs, and the cameraperson stood to the right of the surgeon (near the patient’s left leg) [22]. A 2-cm vertical transumbilical incision was made, and either a SILS port (Covidien Inc., Norwalk, CT, USA) or the hand-made glove method was used [23]. A 5-mm flexible endoscope (Olympus, Tokyo, Japan) was used for intra-abdominal visualization. Cholecystectomy was then performed as mentioned above and as described previously [24,25].

Open cholecystectomy (OC) was performed through a right subcostal or a midline incision. In almost all cases, decompression of the gallbladder was performed using needle aspiration. Dissection was performed to identify the cystic duct and the common bile duct. In cases of advanced local inflammation, the dissection was performed from the fundus towards Calot’s triangle. Intraoperative cholangiography was routinely performed. Finally, the gallbladder was removed from the liver bed.

**Figure 1:**
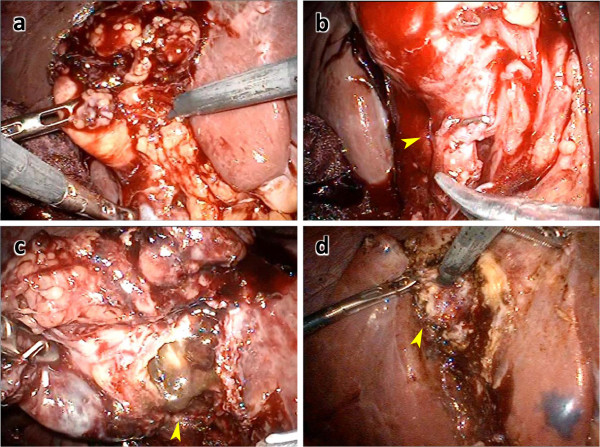
**Representative intraoperative findings of cholecystomucoclasis for acute cholecystitis. a**: Overview of acute cholecystitis. **b**: Double-clipped cystic artery (arrowhead). **c**: The gallbladder was incised carefully because of the lack of a clear indication of the correct layer. **d**: The gallbladder mucosa cauterized by electrocautery (arrowhead).

**Figure 2:**
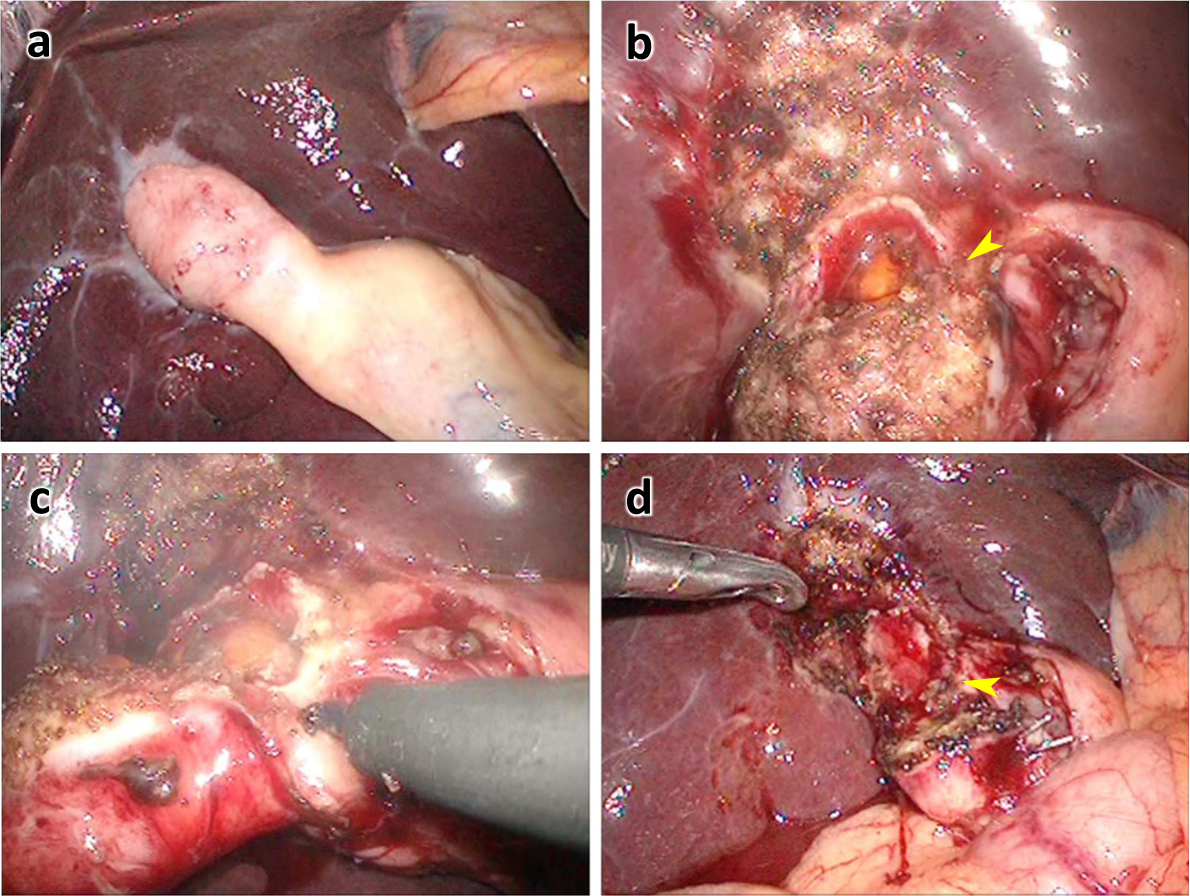
**Representative intraoperative findings of cholecystomucoclasis for atrophic cholecystitis. a**: Overview of atrophic gallbladder in chronic cholecystitis. **b**: Injury of the gallbladder body wall while dissecting from the liver bed. The gallbladder mucosa and gallstones were exposed (arrowhead). **c**: The gallbladder was incised consecutively from the site of the exposed gallbladder mucosa. **d**: Overview after gallbladder removal showing the residual gallbladder posterior wall (arrowhead).

In principle, the gallbladder should be totally resected. However, CM was considered, at the discretion of the surgeon, to prevent massive bleeding or bile duct injury. In particular, CM was applied in cases of a thinned necrotic gallbladder wall due to advanced inflammation, a thickened sclerotic gallbladder wall because of advanced inflammation, the inability to determine the exact orientation of the Calot’s triangle, and the burial of the gallbladder deep within the liver bed. In severe, gangrenous cholecystitis, the dissection began from the fundus to the neck of the gallbladder, after decompression of the gallbladder. The residual gallbladder mucosa was cauterized by electrocautery or argon plasma coagulation. If the gallbladder wall was accidentally damaged, the gallbladder was continuously dissected from the orifice (Figure 1, 2).

### Data collection

The data were retrospectively collected from medical records, operative reports, and histopathological reports. The outcomes for the SC and CM groups were evaluated. The outcome measures included the conversion rate (laparoscopic to open), operation time, loss of blood (low bleeding was defined as 0 mL), maximum preoperative CRP level, American Society of Anesthesiologists physical status (ASA-PS) score, success rate of intraoperative cholangiography, treatment of the proximal bile duct stump, intraoperative complications, postoperative complications, presence or absence of a drain, and length of hospital stay.

### Statistical analysis

The values were expressed as means ± standard deviations (SD). The statistical analysis was conducted using the 2-sided Student’s *t* test and the Mann–Whitney *U* test for continuous data or Fisher’s exact test and the chi-squared test for categorical data. All statistical analyses were performed using the SPSS 10.0 software package (SPSS, Chicago, IL, USA). Significance was defined as *P* < 0.05.

## Results

### Patient characteristics and preoperative findings

The 174 patients who underwent cholecystectomy were divided into the CM and SC groups. Data from the 2 groups are summarized in Table 2. In the CM group, the laparoscopic approach was used for 17 patients, but SILC was not applied. There was no difference in the sex ratio of the 2 groups (*P* = 0.491). Patients in the CM group were, on average, almost 10 years older than those in the SC group (*P* = 0.006). The preoperative general condition was assessed according to the ASA-PS score. The CM group tended to include patients with scores indicating greater severity (*P* = 0.038), and all patients with life-threatening indications (classified as grade 4) underwent SC. Preoperative laboratory data showed a significant difference in CRP levels between the CM and SC groups (18.1 ± 10.5 mg/dL vs. 4.7 ± 8.1 mg/dL, *P* < 0.001).

**Table 2:** Characterization of the cholecystomucoclasis and standard cholecystectomy groups, preoperative findings, and conversion rates

	**CM**	**SC**	***P*value**
	**(n=18)**	**(n=156)**	
Sex			0.491
Male	11	82	
Female	7	74	
Age (years)			0.006
Mean	71.1±8.4	61.9±14.6	
Range	58–84	23–95	
ASA-PS classification			0.038
1	2	37	
2	8	89	
3	8	26	
4	0	4	
Mean CRP in mg/dL	18.1±10.5	4.7±8.1	<0.001
Range CRP in mg/dL	0.41–36.20	0.02–34.04	
Approach to laparotomy			
LC (conventional)	17	128	
SILC	0	18	
OC	1	10	

CRP: C-reactive protein, LC: Laparoscopic cholecystectomy, SILC: Single-incision laparoscopic cholecystectomy, OC: Open cholecystectomy.

### Operative findings

The mean operation time was 129 min (range, 63–190 min) for the CM group and 108 min (range, 50–375 min) for the SC group (*P* = 0.084). The extent of bleeding was not significantly different between the 2 groups (*P* = 0.247). Intraoperative cholangiography was routinely attempted in all cases. The success rate for intraoperative cholangiography was significantly lower in the CM group (50% [9/18]) than in the SC group (92.9% [145/156], *P* < 0.001). When the anatomy of the Calot’s triangle was unclear or inflammation in the gallbladder neck was advanced, the bile duct stump was treated at the neck and the cholecystectomy was limited to a partial resection. The site of the proximal bile duct stump was examined in the 2 groups. The number of patients treated at the neck of gallbladder was significantly more in the CM group than in the SC group (*P* < 0.001). The treatment of the stump of the proximal bile duct was also investigated; a clip was used for almost all patients of the SC group (84.0% [131/156]), whereas various methods (e.g., endoloop, ligation, suture, and elastic yarn) were used for patients in the CM group. In the CM group, the stump could not be closed in 2 patients; thus, only drainage was applied.

Conversion from LC to open cholecystectomy (OC) was usually implemented to prevent bile duct injury within 30 min of beginning the laparoscopy. In some cases (e.g., massive bleeding from the liver bed or identification of bile duct injury after intraoperative cholangiography), OC was used to repair the damage. The conversion rate was significantly higher in the CM group (35.3% [6/17]) than in the SC group (4.8% [7/146], *P* < 0.001) (Table 3).

**Table 3:** Operative findings

	**CM**	**SC**	***P*value**
	**(n=18)**	**(n=156)**	
Operation time (min)			
Mean	129±34	108±48	0.084
Range	63–190	50–375	
Amount of bleeding (mL)			
Mean	146.7±185.2	79.7±236.4	0.247
Range	0–675	0–1970	
Success rate of			<0.001
intraoperative cholangiography			
Possible	9	145	
Not possible	9	11	
Site of proximal bile duct stump			<0.001
Cystic duct	11	148	
Neck of gallbladder	7	8	
Methodology of treatment			
for bile duct stump			
Clip	6	131	<0.001
Endoloop	1	2	
Ligation	4	7	
Suture	3	6	
Elastic yarn (for transcystic drainage)	2	10	
None	2	0	
Conversion rate (%)	6/17(35.3)	7/146(4.8)	<0.001

CM: Cholecystomucoclasis, SC: Standard cholecystectomy.

### Complications and adequacy

**Figure 3:**
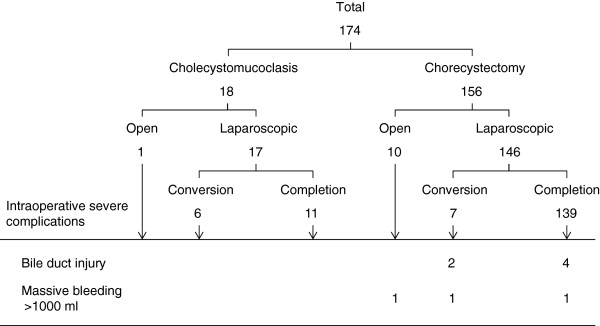
Detailed analysis of the severe intraoperative complications resulting from cholecystectomy.

The intraoperative and postoperative complications are described in Table 4. Although the 2 groups did not differ significantly in this regard (*P* = 0.578), the CM group had no intraoperative complications, whereas the SC group had a relatively high incidence of complications (Figure 3). Three cases of massive bleeding (>1000 mL) and 6 cases of bile duct injury were observed in the SC group.

**Table 4:** Complications, reoperations, additional treatments, drains, and lengths of hospital stay

	**CM**	**SC**	***P*value**
	**(n=18)**	**(n=156)**	
Intraoperative complications			0.578
Total	0	9	
Massive bleeding (>1000 mL)	0	3	
Bile duct injury	0	6	
Postoperative complications			0.001
Total	6	10	
Major intra-abdominal bleeding	0	2	
Bile leakage	2	4	
Wound infection	3	2	
Bacteremia (catheter infection)	0	1	
Retained calculus	1	1	
Reoperation	0	3	0.839
Major intra-abdominal bleeding	0	1	
Bile leakage	0	2	
Additional treatment (EBD, PTCD)	1	4	0.06
Drain			< 0.001
Presence	17	61	
Absence	1	95	
Hospital stay (days)			0.495
Mean	13.2±6.4	8.9±26.1	
Range	5–27	3–330	

CM: Cholecystomucoclasis, SC: Standard cholecystectomy, EBD: Endoscopic biliary drainage, PTCD: Percutaneous transhepatic cholangial drainage.

Postoperative complications were observed in 4 patients of the CM group. Bile leakage and wound infection occurred in 2 patients and a residual calculus, which was subsequently removed endoscopically, was observed in 1 patient. Additional surgical treatment was not required for any of the CM patients. In the SC group, postoperative complications were observed in 10 patients. Two patients experienced intra-abdominal hemorrhage: 1 patient required reoperation and the other was treated conservatively with blood transfusions. Bile leakage was observed in 4 patients: 1 was treated conservatively, 1 needed endoscopic drainage, 1 required immediate reoperation, and 1 required reoperation even though endoscopic drainage and percutaneous transhepatic cholangial drainage were both attempted.

A drain was inserted into the liver bed in almost all CM patients (17/18), but only in some of the SC patients (61/156, *P* < 0.001). The average length of the hospital stay was 13.2 ± 6.4 days (range, 5–27 days) in the CM group and 8.9 ± 26.1 days (range, 3–330 days) in the SC group (*P* = 0.495).

Histopathological examination was performed for all patients, with gallbladder cancer being diagnosed in 2 patients, who received additional treatment. No mortality was associated with the procedures in either group.

## Discussion

Difficulties in performing cholecystectomy include a lack of clarity of the anatomical orientation of the Calot’s triangle, resulting from severe, acute inflammation or chronic atrophic sclerotic change. In this situation, subtotal cholecystectomy is recommended for a safe surgery. Subtotal cholecystectomy can be performed using 2 methods: dissecting the neck of the gallbladder rather than approaching the Calot’s triangle to prevent bile duct injury or not removing the posterior wall of the gallbladder to prevent bleeding from the liver bed. In the latter method, ablation of the remaining mucosa is common and is referred to as CM.

In principle, the gallbladder should be completely resected, as gallbladder carcinoma has been reported in 0.3–1.5% of patients who have undergone cholecystectomy [26,27]; therefore, it is necessary to check for any signs of malignancy during preoperative diagnostic imaging. Because CM or subtotal cholecystectomy may result in an intraoperative bile leak into the abdominal cavity, these procedures are associated with potential dissemination. Although there are no reports of residual gallbladder cancer following CM or subtotal cholecystectomy, Shimizu et al. reported a case of biliary tract cancer in the liver bed after subtotal cholecystectomy. They considered the possibility of a peripheral type of intrahepatic cholangiocarcinoma as well as carcinomas from the residual gallbladder mucosa or the Luschka duct [28]. Therefore, regular follow-ups with diagnostic imaging are needed, even when the patient does not show pathological evidence of malignancy.

The frequency of postoperative complications in subtotal cholecystectomies has been reported to be 6.7–20.7% [17-21], with the patients having an average age of 53–62.9 years. In the current study, a relatively high incidence of postoperative complications (33% [6/18]) was observed, and the average age of patients in the CM group was 71.1 ± 8.4 years. In particular, 2 patients were found to have postoperative bile leakage, both of whom were treated with drainage only, without processing of the bile duct stump. One patient required endoscopic biliary drainage (EBD) and the other recovered spontaneously without any additional treatment. The reason for the spontaneous closure was presumed to be the result of postoperative firm adhesion and scar formation, which is expected in patients with advanced inflammation. The SC group had 4 patients with postoperative bile leakage. Two patients required a reoperation for bile leakage closure, and the other 2 were treated by diversion of bile from the leakage site by EBD or percutaneous transhepatic cholangial drainage. There were no significant differences between the two groups in additional treatment (*P* = 0.06), but the treatment for bile leakage in SC group was more difficult.

Few detailed studies have reported on intraoperative findings in these types of cholecystectomies. In the CM group, there was no evidence of intraoperative damage to the biliary tract, a finding that was similar in the SC group. Additionally, there was no significant difference in the amount of bleeding between the 2 groups, and the CM group did not include patients exhibiting massive bleeding (>1000 mL). During cholecystectomy, bleeding primarily originates from the cystic arteries and the liver bed; CM and subtotal cholecystectomy help prevent bleeding from these important locations.

The conversion rate to OC was 35.3% in the CM group, which was higher than that in previous reports (1.7–7.7%) [18.19.21]. Thus, we emphasize on safety in our surgical procedures and convert to OC without hesitation in difficult cases. The lack of intraoperative complications in the CM group is probably the result of these efforts. Furthermore, patients who needed reoperation were observed only in the SC group, highlighting the need for a flexible approach according to patient characteristics rather than adherence to a particular policy of laparoscopic surgery in order to avoid unnecessary intraoperative complications and conversion to laparotomy.

OCs are typically more closely associated with advanced age, poor general condition, or a high inflammatory response than are laparoscopic surgeries [29]. These same characteristics are also associated with an increased severity of acute cholecystitis, which may result in a more difficult surgery. The aforementioned patient characteristics are collective, but not independent, risk factors for postoperative complications of acute gangrenous cholecystitis [30,31]. In addition, Schäfer et al. have reported that advanced age and a high serum CRP level may be predictive factors for the surgical procedures that are used in patients undergoing laparotomy and patients who were converted to laparotomy [32]. The current study was a retrospective study, with a selection bias based on intraoperative findings; the CM group showed an advanced age and a high serum CRP level. With the transition to CM and open surgery in mind, the conversion to a safe surgical procedure should be considered in elderly patients with high inflammation of the gallbladder neck.

## Conclusions

The intraoperative technical characteristics of SC and CM were analyzed, and a low rate of handling of the cystic duct stump and a low success rate for intraoperative cholangiography were observed in the CM group. However, no significant differences were observed in the operative time, amount of bleeding, and number of intraoperative complications. Compared with SC, high conversion rates and drain insertion rates were noted in patients undergoing CM, but the length of hospital stay did not differ significantly. CM is thus considered to be a safe and valid surgical procedure. In addition, surgeons should not hesitate to transition to CM for patients with advanced age, poor general condition (high ASA classification), and a high serum CRP level.

## Competing interests

The authors declare that they have no conflicts of interest.

## Authors’ contributions

TT performed most of the experiments, participated in the design of the study, performed the statistical analysis, and drafted the manuscript. TO was involved in the study design and coordination. TM, SS, and TN participated in the design of the study and helped draft the manuscript. All authors have read and approved the final manuscript.

## Pre-publication history

The pre-publication history for this paper can be accessed here:

http://www.biomedcentral.com/1471-230X/12/113/prepub
